# From Threat to Opportunity: Harnessing the Invasive *Carpobrotus edulis* (L.) N.E.Br for Nutritional and Phytotherapeutic Valorization Amid Seasonal and Spatial Variability

**DOI:** 10.3390/md21080436

**Published:** 2023-08-01

**Authors:** Catarina Guerreiro Pereira, Nuno R. Neng, Luísa Custódio

**Affiliations:** 1 Centre of Marine Sciences CCMAR, Faculty of Sciences and Technology, Ed. 7, Campus of Gambelas, University of Algarve, 8005-139 Faro, Portugal; cagpereira@ualg.pt; 2 Centro de Química Estrutural, Institute of Molecular Sciences, Departamento de Química e Bioquímica, Faculdade de Ciências, Universidade de Lisboa, Campo Grande, 1749-016 Lisboa, Portugal; ndneng@fc.ul.pt; 3 Laboratório de Ciências Forenses e Psicológicas Egas Moniz, Molecular Pathology and Forensic Biochemistry Laboratory, Centro de Investigação Interdisciplinar Egas Moniz, Egas Moniz School of Health and Science, Campus Universitário, Quinta da Granja, Monte de Caparica, 2829-511 Caparica, Portugal

**Keywords:** Hottentot-fig, invasive plants, nutrients, phenolics, antioxidant, anti-inflammatory

## Abstract

*Carpobrotus edulis* (L.) N.E.Br. (Hottentot-fig) is a problematic invasive species found in coastal areas worldwide. Mechanical removal is a common control method, leaving the removed biomass available as a possible source of natural phytochemicals with prospective commercial applications. While the Hottentot-fig’s vegetative organs have been studied previously, this work establishes for the first time a seasonal and spatial comparative analysis of its nutritional, chemical, and bioactivity profiles (in three locations over four seasons). Proximate and mineral contents were assessed, along with its phenolic composition and in vitro antioxidant and anti-inflammatory properties. Hottentot-fig’s biomass offered a good supply of nutrients, mainly carbohydrates, proteins, and minerals, with a tendency for higher concentrations of the most relevant minerals and proteins in autumn and winter, and in plants from sites A (Ria de Alvor lagoon) and B (Ancão beach). The extracts were rich in polyphenolics, with higher levels in spring and summer, especially for luteolin-7-*O*-glucoside and salicylic and coumaric acids. The extracts were also effective antioxidants, with stronger radical scavenging activities in spring and summer, along with anti-inflammatory properties. Our results suggest that the usually discarded plant material of this invasive halophyte could be valuable as a source of natural products with potential biotechnological applications in the food and nutraceutical industries.

## 1. Introduction

Coastal environments act as transitional zones between land and sea ecosystems. They harbor specialized fauna and flora while providing crucial ecological services, such as water filtration, flood control, and storm protection, but they are highly vulnerable to natural and human-induced changes [1,2,3]. One such change is the presence of invasive species that results in detrimental effects on ecosystem functioning and services, leading to a significant loss of biodiversity and the suppression of native species [4]. Additionally, the economic costs of biological invasions are substantial, with estimates based on models suggesting that in 2020, at least EUR 18.3 billion was required across Europe for reliable observed costs alone [5]. Moreover, managing invasive species is challenging and expensive, with overall costs reaching billions of Euros annually [6].

The phytochemical composition of invasive plants must be diverse and with high bioactive potential to endow them with coping mechanisms to adapt to new habitats, particularly regarding competitive advantage (e.g., allelopathy) and resistance to predators (e.g., deterrents) [4]. Furthermore, their rapid growth characteristics make them promising candidates for sustainable utilization as feedstock in diverse industries. Harnessing the biotechnological potential of biomass from invasive species is, therefore, considered a valuable strategy since it not only helps mitigate the negative impacts of these plants but also enables the production of beneficial bioactive products with potential commercial applications [4,7].

*Carpobrotus edulis* (L.) N.E.Br. (Aizoaceae), commonly known as ice plant, sour fig, or Hottentot-fig, is a South African native plant inhabiting coastal areas in many parts of the world, including most Mediterranean countries. It can be found on coastal rocks, sand dunes, and sea cliffs, spreading widely while displacing several native species, and is considered a problematic invasive species with negative impacts on the biodiversity of the invaded ecosystems [8,9]. This perennial succulent halophyte has been introduced in all five continents for ornamental and soil stabilization purposes but has proved to be an aggressive competitor of local flora, one that is able to change soil physicochemical properties and geochemical processes [4,9,10]. Traditionally, Hottentot-fig has been considered a medicinal and edible plant. It has been used to treat a variety of ailments, including inflammation, dysentery and diarrhea, tuberculosis, diabetes, sinusitis, blood pressure, coughs and colds, throat and mouth infections, digestive problems, and skin conditions including irritation, wounds, chilblains, eczema, and burns, has been employed as an antiseptic, and even to manage common infections in HIV patients [4,9]. Additionally, it is consumed raw or used in jams, jellies, and other preserves; its leaves are used as a vegetable, either raw or cooked, and its fruits have a sweet, tangy flavor. There are also reported uses as a food additive (for flavoring) and preservative (antimicrobial) [9,10].

Moreover, Hottentot-fig has been shown to be a source of bioactive compounds by various studies that credit it with many bioactivities, such as in vitro antioxidant and anti-melanogenic (e.g., reported in water, ethanol, and acetone extracts from the fruits’ peel and flesh) [11], wound-healing and anti-skin aging (e.g., aqueous extracts from leaves) [12], antibacterial and anti-fungal (e.g., in hydro-ethanolic and aqueous extracts from flowers) [13,14], and neuroprotective (e.g., hexane, dichloromethane, ethyl acetate, and methanol extracts from leaves) [15,16], to name some properties. Among the described phytoconstituents present in this halophyte, flavonoids and phenolic acids are usually predominant, along with tannins, coumarins, triterpenes, procyanidins, and alkaloids, among others [9,11,12,13,14,15,16]. These groups of bioactive molecules are potentially responsible for the medicinal properties attributed to this plant.

**Figure 1:**
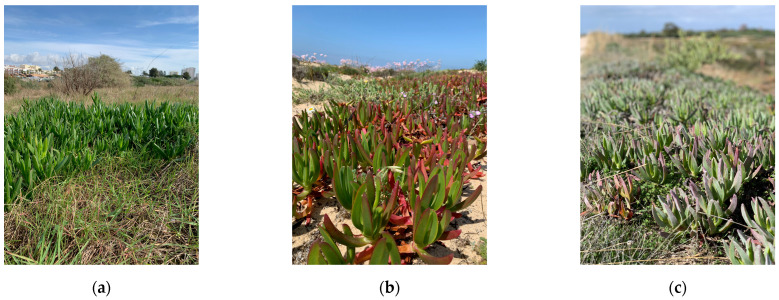
*Carpobrotus edulis* (Hottentot-fig) in southern Portugal: (**a**) Ria de Alvor lagoon (winter); (**b**) Ancão beach (spring); (**c**) Ria Formosa lagoon (autumn). Photos by Catarina Guerreiro Pereira.

**Figure 2:**
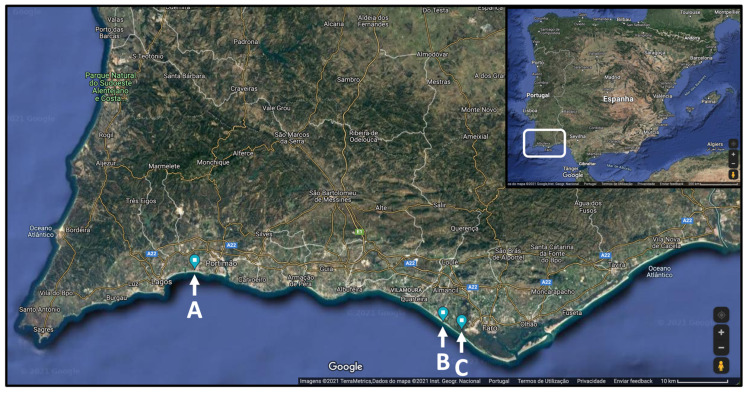
Locations of *C. edulis* (Hottentot-fig) collection in southern Portugal: (A) Ria de Alvor lagoon, (B) Ancão beach, (C) Ria Formosa lagoon. Adapted from Google Maps^®^ 2021.

While Hottentot-fig has been the focus of various studies concerning its bioactivities and chemical composition, only Chokoe et al. [17] considered spring versus autumn while analyzing antibacterial activity. Seasonal and spatial analysis have not been considered, to the best of our knowledge. This information can be useful to characterize this invasive plant targeting its potential biotechnological applications, while establishing seasonal and location profiles for optimizing the exploration of discarded plant material. Hence, this work establishes a comparative analysis of nutritional, chemical, and bioactivity profiling of Hottentot-fig samples from three different locations in southern Portugal, throughout the four seasons of the year (Figure 1 and Figure 2). The locations selected (Figure 2) correspond to three “hotspots” of high biomass density of Hottentot-fig on the Algarve coast: two lagoons of different characteristics (~70 km apart) and one beach (adjacent to the bigger lagoon). Proximate and mineral compositions were assessed, along with their phenolic composition (spectrophotometrically and by High-Performance Liquid Chromatography-Diode Array Detection (HPLC-DAD)) and in vitro antioxidant and anti-inflammatory properties. 

## 2. Results and Discussion

### 2.1. Nutritional Profiling

**Figure 3:**
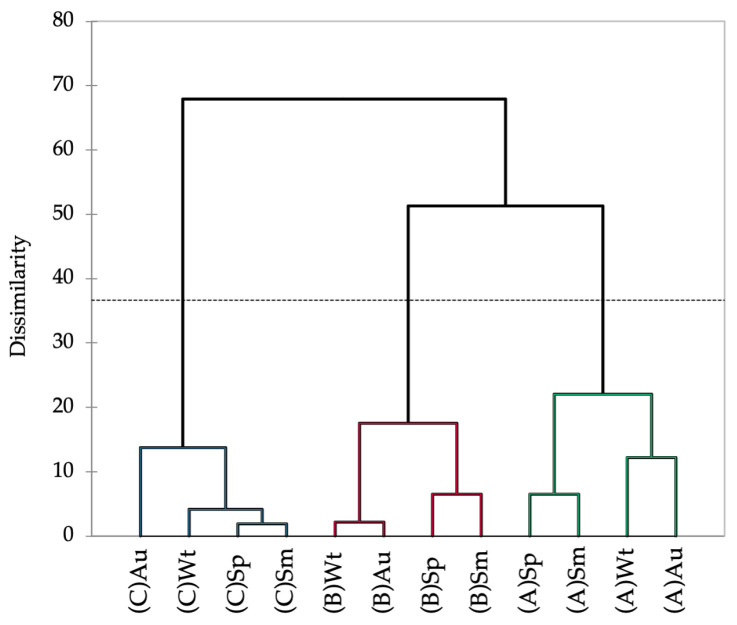
Dendrogram representing the Agglomerative Hierarchical Clustering of the seasonal and spatial nutritional profile (proximate composition and mineral content) of *C. edulis* (Hottentot-fig) aerial parts. (A) Ria Alvor, (B) Ancão beach, (C) Ria Formosa. Wt: winter, Sp: spring, Sm: summer, Au: autumn.

The Hottentot-fig’s nutritional composition varied significantly between seasons and locations (Table 1). The levels of moisture, ash, crude protein, and crude fat showed an overall trend of higher values in autumn and winter, and levels were also higher in plants from site A. Conversely, carbohydrates and fiber showed higher levels in spring and summer, with levels being higher in plants from site A for fiber and from site B for carbohydrates. Such a pattern seems less apparent for minerals, although a tendency for higher concentrations of Ca, Mg, Na, Fe, Mn, and Zn in autumn and winter is discernible. Plants from sites A and B have higher levels overall of Ca, Na, and Fe, but the plants from site C showed greater amounts of the other minerals. The association between spring and summer, and between autumn and winter, is depicted in the dendrogram of the seasonal and spatial nutritional profile of Hottentot-fig (Figure 3). The dotted line in Figure 3 represents the automatic truncation, leading to three clusters; within each cluster, spring and summer are more closely linked, as are autumn and winter in the second and third clusters. Additionally, each cluster groups the data according to location, implying that spatial variability may have more impact on the nutritional profile of this plant than season variability, as the data are more closely linked by location (across seasons) than by season (across locations).

The observed seasonal and spatial variability in the nutrient content of Hottentot-fig may have implications for its potential uses. The nutritional profile of halophyte plants may change with environmental fluctuations as a strategic adaptation to preserve intracellular ionic balance, avoiding excessive salt accumulation and protecting cellular structures from excessive oxidative stress [18]. For example, the accumulation of carbohydrates contributes to osmotic adjustment [18,19]; thus, a higher carbohydrate concentration in spring and summer could be a strategy to cope with salt stress, which can increase in periods of higher temperatures and lower rainfall, as was the case in these seasons (Table 2). Moreover, higher carbohydrate levels in plants from site B, a beach, could imply higher stress in such a location than in the other sites (lagoons). On the other hand, higher values of moisture, ash, protein, fat, and most minerals in autumn and winter could be attributed to favorable meteorological conditions (e.g., higher humidity and rainfall, or lower salinity) that are usual in these seasons (Table 2), which allow their uptake and subsequent allocation for growth. In fact, some authors report that protein content decreases with increased salinity, which is typical in hotter seasons such as spring and summer (as shown in Table 2) [18,20]. Other factors, such as the plant developmental/growth stage, may also influence nutrient content changes throughout the year [21,22]. For instance, fiber content changes over the seasons according to growth stage and temperature, being lower in cool weather (associated with vegetative states) and increasing with warmer temperatures [23]. This was the case for the fiber content of Hottentot-fig, with higher levels in the warmer spring and summer seasons (as depicted in Table 2). As for spatial variability, soil composition can vary widely between locations. Sites A and C are in lagoons with different characteristics and are of considerably different sizes (site A: ~15 km^2^ small shallow estuary [24]; site C: ~100 km^2^ multi-inlet barrier system [25]), while site B is a sandy beach. These differences are probably reflected in the soil composition of each location and, since soil properties are the major influence on plant nutrient contents [26], that fact alone may explain the differences in the nutrient contents of Hottentot-fig from the distinct sites. Reports on the Hottentot-fig’s nutritional composition are scarce, and, to the best of our knowledge, no other studies have been published concerning seasonal and spatial analyses of its nutritional profile. However, an analogous study with the halophyte *Cladium mariscus* [27] showed some similarities in the seasonal variation of nutrient content, such as higher levels of ash in autumn and of fiber in summer, which are possibly related to the plants’ reproductive stages.

Hottentot-fig is a succulent halophyte [29], its succulence (specialized water-storing tissues) being an important characteristic for surviving in harsh conditions, such as drought and high salinity [30]. Therefore, high levels of moisture in the Hottentot-fig’s aerial parts (85.2–94.3 g/100 g, Table 1) were expected and were comparable to those obtained in leaves of this species (92.3 g/100 g) [15], while they were higher than reported in its fruits (77.6 g/100 g) [31]. In most edible vegetables, the moisture content is above 80%, and is sometimes more than 90%, as in spinach, cabbage, or lettuce [32]. Higher moisture levels make vegetables more appealing to consumers as it is perceived as showing freshness [32,33]. Ash content is associated with the minerals that plants retain and is largely correlated with salts concentration in the soil, meaning that halophytes are rich in this nutrient [33]. This is corroborated by the present findings (18.6–27.5 g/100 g, Table 1), wherein values are similar to those found in different Hottentot-fig organs (21.4–32.0 g/100 g) [13,15], but are higher than in its fruits (10.1 g/100 g) [31]. Despite being rich in ash, levels in different halophyte species can vary from lower contents, such as 10.5 g/100 g in *Atriplex nitens* [34], to higher contents, such as 43.3 g/100 g in *Sarcocornia fruticosa* [35]. Comparatively speaking, Hottentot-fig’s ash contents can be considered high, even more so when compared to most vegetables, where the ash content usually ranges between 5 and 10 g/100 g [36], as found in tomatoes (7.1 g/100 g) or potatoes (10.4 g/100 g), or the ash-rich spinach (17.3–22.3 g/100 g) [37]. Crude protein, an estimation of the protein content in the samples, ranged between 1.6 and 6.2 g/100 g (Table 1), values similar to those in other reports for the Hottentot-fig’s organs (4.6–5.4 g/100 g) [13,15]; its fruits, however, are described as a much better protein source at 23.5 g/100 g [31]. Likewise, other halophytes have been considered good protein sources, with values varying between 5 g/100 g (*Climacoptera lanata* or *Salicornia ramosissima*) and 13 g/100 g (*Arthrocnemum macrostachyum* or *Atriplex nitensi*) [33,38], much like the values for regular edible vegetables, which can range from 0.65 g/100 g (cucumbers) to 5.42 g/100 g (peas) [32]. Vegetables typically possess low levels of fat [32], but the crude fat content in the Hottentot-fig’s aerial parts was particularly low, varying between 0.6–1.3 g/100 g (Table 1), in comparison to those described in the Hottentot-fig’s leaves, flowers, and fruits (1.7–4.4 g/100 g) [13,15,31]. While halophyte species can contain higher crude fat levels, as, for example, 6.9–7.8 g/100 g for *Mesembryanthemum nodiflorum* [35], most common vegetables contain less than 1% fat, namely 0.12 g/100 g in asparagus or 0.39 g/100 g in spinach [32], which is lower than was currently found in the aerial parts. Conversely, carbohydrate contents were high (66.2–78.5 g/100 g, Table 1), as they are usually the second most abundant component after water [32], but carbohydrates were estimated by difference, which may have caused an overestimation of its contents. Nevertheless, the recorded values are analogous to those described for different Hottentot-fig’s organs (61.4–70.2 g/100 g) [13,31] and other halophytes, such as *Crithmum maritimum* (69.5 g/100 g) [39] or *Juncus maritimus* (73 g/100 g) [40], although they were higher than in most vegetables (e.g., leek at 14.2 g/100 g or yam at 27.9 g/100 g) [32]. As for fiber, estimated in terms of neutral detergent fiber (NDF), comprising levels of cellulose, hemicellulose, and lignin [41], it is considered a good indicator of feed quality and is used as an assessment of insoluble dietary fiber [42]. The values for the Hottentot-fig’s aerial parts (17.1–26.0 g/100 g, Table 1) are slightly lower than those reported for its leaves (30.4 g/100 g) [15], but overall, the results are akin to levels found in other halophytes, such as *Salicornia* and *Sarcocornia* species (20.8–22.5) [33], or other vegetables, such as carrot (18.2 g/100 g) or cauliflower (21.3 g/100 g) [43].

Hottentot-fig’s mineral content (Table 1) was characterized by a greater abundance of Na and Ca within the macro elements, and of Mn, Zn, and Fe in the trace minerals. A characteristic feature of halophyte plants is their high Na content, a reflection of the environment in which these plants live [29], with Na content being reported as high as 30 g/100 g DW (in cultivated *Sarcocornia fruticosa*) [35]. Hottentot-fig’s Na content varied between 2.3 and 4.8 g/100 g, values akin to those reported in its leaves (4.4 g/100 g) [15], but lower than that reported for halophytes such as *Salicornia* species (9–17 g/100 g) [33,44,45]. Nevertheless, values were higher than for most vegetables (max. 1.1 g/100 g in canned sweetcorn) [46], although they were akin to those in some foods, such as canned anchovies (4 g/100 g) [46] or seaweed products (3.96 g/100 g) [47]. Interestingly, the Hottentot-fig’s aerial parts are a good source of Ca, displaying values of between 2.5 and 4.3 g/100 g, similar to previously reported levels (leaves 2.7 g/100 g) [15]. These levels are higher than those described for cultivated halophytes such as *S. fruticosa*, *Suaeda maritima*, and *M. nodiflorum* (1.8, 1.6, and 1.9 g/100 g, respectively) [35], and higher than for Ca-rich vegetables such as arugula, watercress (1.93 and 2.45 g/100 g) [48], or spinach (0.06–0.6 g/100 g) [32]. As for the most abundant trace elements in the Hottentot-fig’s aerial parts, Fe (2.3–5.3 mg/100 g), Mn (2.3–24.8 mg/100 g), and Zn (1.1–8.7 mg/100 g) levels were akin to those described in Hottentot-fig leaves (Fe 8.0 mg/100 g, Mn 6.6 mg/100 g, Zn 2.0 mg/100 g) [15]; Mn and Zn levels were also within the same range as those reported for the above-mentioned halophytes (Mn 3.1–22.1 mg/100 g; Zn 2.1–9.8 mg/100 g) [35], although they were lower for Fe (46–339 mg/100 g) [35]. Vegetables such as beans, peas, chickpeas, lentils, or spinach are excellent sources of Fe (1.0–11.1 mg/100 g), Mn (0.2–2.6 mg/100 g), and Zn (0.2–4.3 mg/100 g) [46], the levels of which are comparable to those determined in the Hottentot-fig’s aerial parts. Moreover, potentially toxic elements such as Ni, Cd, and Pb were either below the reference value for tolerable upper intake levels (Ni, 1 mg/day) [49], below the legislated value (0.3 mg/kg wet weight for Pb) [50], or were not detected (Cd, below LOQs; Table 1).

Overall, this invasive plant’s aerial parts show adequate nutritional potential, akin to those from regularly consumed vegetables and edible halophytes, particularly as a source of carbohydrates, proteins, and minerals such as Na, Ca, Fe, Mn, and Zn. The best season and location for optimized nutrient exploration of the discarded plant material was not clear, but a tendency was noticeable for higher concentrations of the most relevant minerals and protein in autumn and winter and in plants harvested from sites A and B.

### 2.2. Chemical Profiling

**Figure 4:**
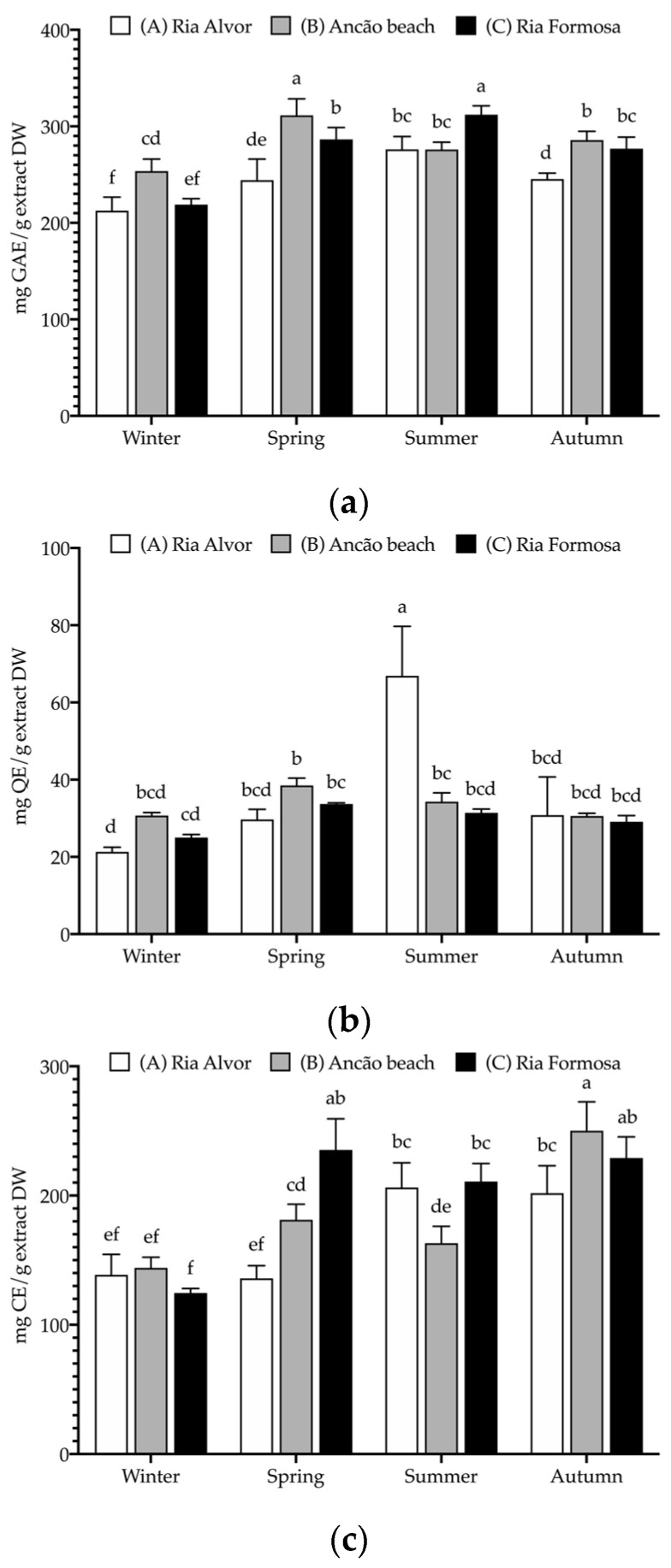
Seasonal and spatial polyphenolic contents (mg/g extract DW (dry weight)) of *C. edulis* (Hottentot-fig) extracts: (**a**) total phenolic content (TPC, mg GAE/g DW); (**b**) total flavonoid content (TFC, mg QE/g DW); (**c**) condensed tannin content (CTC, mg CE/g DW). Data represent the mean ± SD (*n* = 6). For each phenolic group, different letters represent significant differences (*p* < 0.05). GAE—gallic acid equivalents, QE—quercetin equivalents, and CE—catechin equivalents.

The seasonal and spatial phenolic composition of Hottentot-fig extracts were assessed in terms of their phenolic groups, namely, total phenolics (TPC), flavonoids (TFC), and condensed tannins (CTC); the results are depicted in Figure 4. 

Hottentot-fig extracts presented high levels of all phenolic groups, particularly of TPC, which was above 200 mg GAE/g extract DW (GAE—gallic acid equivalents) in all locations and seasons, considering the threshold of 20 mg/g that many authors apply to deem an extract rich in phenolic contents [51,52,53]. Higher TPC values were noted in spring (site B; 311 mg GAE/g) and summer (site C; 312 mg GAE/g), while the lowest values were registered in winter (all sites; 213–254 mg GAE/g). These values fall within the wide range (6–378 mg GAE/g extract) reported by other authors for various plant organs and extract solvents [11,12,13,15,54]. Tannins were also exceptionally high, varying from 125 to 250 mg CE/g extract DW (CE—catechin equivalents), especially when compared with other studies: 58–101 mg RE/g in its leaves (hexane, dichloromethane, ethyl acetate, and methanol) [15], or 0.4–20.3 mg CE/g in Hottentot-fig’s fruits (water, ethanol, and acetone extracts) [11]. Following a similar trend to TPC, CTC values were higher in spring (site C; 235 mg CE/g) and autumn (sites B and C; 250 and 229 mg CE/g, respectively), and lower in winter (all sites; 125–144 mg CE/g). As for TFC, except for the higher content in summer (site A; 67 mg QE/g; QE—quercetin equivalents), no discernible trend was evident, and values were lower than described for leaves (298–587 mg CE/g) [12,15], but higher than those in fruits (0.01–1.6 mg QE/g) [11]. Seasonal/location variability on polyphenolic composition would be expected, considering that phenolic content in plants can be influenced by factors such as weather, geography, and environmental stressors, in addition to the effect of organ/tissue and extraction methods [51,55]. This can also help explain the differences in phenolic groups contents between the current results and those of other studies. Increased levels of total phenolics in spring and summer could result from the environmental challenges that are typical of these seasons (e.g., high salinity, UV exposure, drought, or elevated temperatures, as can be seen in Table 2) that induce the plant’s production of these secondary metabolites as a coping strategy. For example, Parida et al. [20] describe significant increases in the levels of total polyphenols with increasing salinity. The same factors could account for location variations, as plants from sites B and C showed higher levels of polyphenols; these locations are closer in proximity, thus potentially experiencing more similar environmental challenges. Additionally, during spring, the aerial parts collected included the flowers (see Figure 1b), which could imply possible organ-related variations. Still noteworthy is the extracts’ high tannin content as tannins hold important potential applications, namely, a role in sensory taste perception [56] and the prevention of cardiovascular disorders or other chronic diseases, such as diabetes mellitus [57].

**Figure 5:**
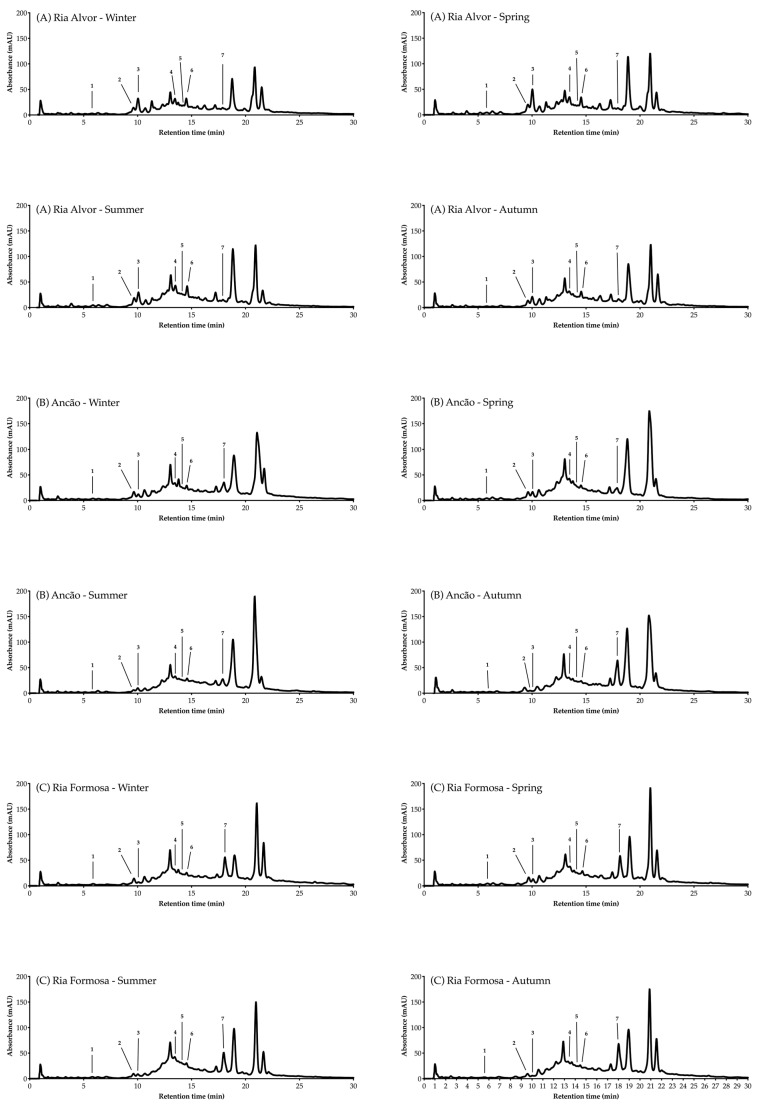
HPLC-DAD analysis (320 nm) of *C. edulis* (Hottentot-fig) extracts throughout the seasons and locations. Peak numbers refer to the compounds in Table 3.

**Figure 6:**
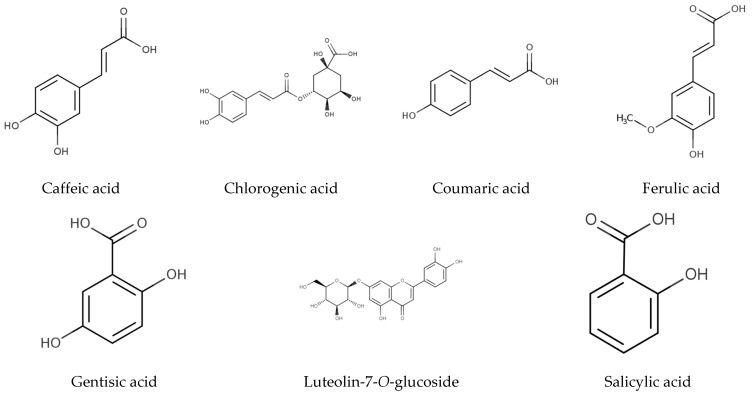
Chemical structure of identified molecules in *C. edulis* (Hottentot-fig) extracts (Table 3; adapted from Phenol-Explorer 2023 [58]).

The individual phenolic compounds present in Hottentot-fig’s extracts throughout the seasons and sites were also identified and quantified by HPLC-DAD, as summarized in Table 3 (chromatograms in Figure 5). Out of the seven compounds identified, six were phenolic acids, namely gentisic, salicylic, chlorogenic, caffeic, coumaric, and ferulic acids, and one was a flavonoid, luteolin-7-*O*-glucoside (molecular structures in Figure 6).

In agreement with the results of the TPC, the levels of individual phenolics were higher in spring and summer, although their sum was increased in autumn for sites B and C, mostly due to higher concentrations of the flavonoid being detected. In fact, luteolin-7-*O*-glucoside, together with salicylic acid, were the most abundant compounds in the samples from sites B and C, while in site A, the most abundant were salicylic and caffeic acids, together with the flavonoid. The prevalence of these compounds may be rooted in the role that phenolic compounds play in plants exposed to harsh environmental constraints, as is the case with halophytes. Luteolin derivatives seem to predominate in plants under full sun, as is usually the case with Hottentot-fig, and is even more predominant in tissues exposed to greater sunlight irradiance, likely serving photoprotective roles [59]. Luteolin-7-*O*-glucoside is also reported to accumulate in response to soil salinity [59], which might contribute to its predominance in the current Hottentot-fig’s samples; it was also a main compound found in Hottentot-fig from Tunisia (water and aqueous-ethanol extracts from leaves) [54]. Salicylic acid is linked to stress tolerance in plants, namely, their adaptation to saline stress [60], having been recognized not only as a regulatory response to abiotic stress but also as a defensive reaction for plant immunity and a hormone in growth and development regulation [61]. It was also found as a major phenolic in Hottentot-fig collected near location C (in the ethyl acetate extracts from leaves) [15]. Caffeic acid is also involved in the plants’ mechanisms of stress tolerance; for example, its higher concentrations increase salt tolerance in plants under salinity stress, enhances plant growth when under drought stress, and improves resistance to stress overall via antioxidant activity [62]. Similarly to these two phenolic acids, gentisic acid has been reported as a signaling molecule in plant defense [63] and in response to salinity stress [64]. Furthermore, several polyphenols are overall involved in cell wall thickening, which confers drought tolerance by mechanically reinforcing the plant tissues and boosting resistance to oxidative stress, which are both key features in the endurance of halophyte plants [59]. For example, ferulic acid is a component of plant cell walls: it binds to lignin, polysaccharides, and proteins, acting as a universal connector between the cell wall polymers [65]. Coumaric acid is also an essential cell wall component, being an important precursor to lignin and other polyphenols [66]. Moreover, in stressful environments, strong antioxidants such as chlorogenic acid are produced to grant the plant protection against oxidative stress; this phenolic acid is also considered a facilitator in the abiotic stress-response mechanisms of plants [67,68].

Besides their plant functions, the major phenolics detected are pharmacologically described with relevant therapeutic bioactivities. Luteolin-7-*O*-glucoside has demonstrated anti-inflammatory, antioxidant, antidiabetic, metabolite-modulatory, antimicrobial, and anticarcinogenic activities [69,70,71]; it has been suggested that it is bioavailable when circulating in the organism, without being metabolized to its conjugates in the gastrointestinal tract [72], which alters the physicochemical properties of flavonoids and can affect their biological activity [73]. Salicylic acid has documented anti-inflammatory, analgesic, antiseptic, antipyretic, antimicrobial, comedolytic, and keratolytic/desmolytic properties [74,75]. Caffeic acid is pharmacologically described as a potent antioxidant, with antimicrobial, anti-inflammatory, neuroprotective, and anticarcinogenic activities [76,77,78]. Most of these activities have also been attributed to the remaining molecules identified, namely, antioxidant and anti-inflammatory (ferulic, chlorogenic, coumaric, and gentisic acids) [65,66,79,80], anti-carcinogenic and neuroprotective (ferulic, coumaric, and gentisic acids) [65,66,80], antidiabetic (ferulic, chlorogenic, and coumaric acids) [65,66,79], or antimicrobial properties (chlorogenic, coumaric, and gentisic acids) [66,79,80].

Other phenolic compounds that have been reported in Hottentot-fig [9,11,12,13,14,15,54] were not herein identified, while gentisic acid is, to the best of our knowledge, currently described in Hottentot-fig for the first time. Variations in the phenolic profile of extracts of a same species are likely due to different geographical, climatical, and/or genetic conditions, although the influence of different methodological procedures, such as sample preparation, extraction, and analysis should not be discarded. Nevertheless, it is worth mentioning that not all compounds in the extracts were identified, as can be seen in the chromatograms in Figure 5, due to the complexity of the extracts’ mixtures.

### 2.3. Bioactivity Profiling

The seasonal and spatial variations in terms of the antioxidant activity of Hottentot-fig extracts are presented in Table 4. Considering the multifaceted aspects of antioxidants and their consequent reactivity, the extracts were assessed for radical scavenging and metal chelating activity using complementary in vitro methods, namely, radical-based (scavenging activity against radicals DPPH• [1,1-diphenyl-2picrylhydrazyl], ABTS•+ [2,2′-azino-bis(3-ethylbenzothiazoline-6-sulfonic acid)], and NO (nitric oxide)) and metal-related (reducing iron capacity (FRAP), and chelating on iron (ICA) and copper (CCA)) assays.

Hottentot-fig extracts were overall effective radical scavengers as almost all samples showed EC_50_ values that were significantly lower than those of the positive controls for the three tested radicals (DPPH•, EC_50_ (BHA) = 0.6 mg/mL; ABTS•+, EC_50_ (BHA) = 0.33 mg/mL; NO, EC_50_ (ascorbic acid) = 1.71 mg/mL; Table 4). The higher activities toward DPPH• and ABTS•+ had EC_50_ values ranging between 0.05 and 0.11 mg/mL in spring and summer for all locations (also in winter for sites A and B, for ABTS•+), while the stronger NO scavenging was between 0.18 and 0.26 mg/mL (IC_50_ values in spring for all locations, plus winter in site C; Table 4). Other authors reported divergent radical scavenging activity (RSA) of this invasive halophyte. Against DPPH•, the leaves’ extracts were either active only at > 1 mg/mL (in methanol and ethyl acetate extracts) [15], had 20–50% activity at 0.1 mg/mL (in water and aqueous ethanol extracts) [54], or showed IC_50_ values of 0.11 mg/mL (in water extract) [12] and 0.02 mg/mL (in aqueous-ethanol extract) [13]. Regarding ABTS•+, the RSA of extracts from Hottentot-fig’s fruits varied with EC_50_ values of 0.56 to 6.4 mg/mL (in water, ethanol, and acetone extracts) [11], while against NO, the leaves’ extracts showed 20–50% activity at 0.1 mg/mL (in water and aqueous ethanol extracts) [54]. Notwithstanding the diverse reports in the literature, the current RSA results were comparable to the best ones described by other authors. The extracts were also strong iron reducers since samples with the highest activity (EC_50_ = 0.10–0.29 mg/mL in spring and autumn for all sites) were as potent as the positive control BHA (EC_50_ = 0.16 mg/mL; Table 4). Comparatively, the water extracts from leaves showed stronger activity (EC_50_ = 0.03 mg/mL) [12], while the methanol extracts were weaker (45% at 1 mg/mL) [15]. As for the chelating properties of Hottentot-fig extracts, they were moderate for copper, compared with the positive control EDTA (EC_50_ = 0.16 mg/mL), and were low for iron (EC_50_ > 10 mg/mL; Table 4). The best samples in CCA showed EC_50_ values from 1.84 to 1.99 mg/mL (spring in sites B and C; Table 4), which are comparable to those obtained with hexane and dichloromethane extracts from leaves (45–50% activity at 1 mg/mL) [15]. Differences between the present results and the literature reports on the antioxidant activity of Hottentot-fig can most likely be pinpointed to the use of different extraction solvents/methods and/or a different sampling season, not discarding environmental variations, all these being factors that can influence the antioxidant profile of natural extracts [81].

Overall, there was a clear tendency toward higher RSA in spring and summer and higher metal-related capacities in spring and autumn for all locations. Conversely, RSA in autumn and metal-related capacities in winter were weaker. As for spatial variation, there is no significant discrimination between locations, which is an opposite pattern to that observed for the nutritional profile, where spatial variability seemed to have more impact (Figure 3). Nevertheless, the closer association of spring and summer, and of autumn and winter, seemed to be constant, having been depicted throughout the nutritional, chemical, and bioactivity profiles. The higher antioxidant activity noted in spring and summer can probably be attributed to the higher phenolic content that was also perceived in this period, considering that the relationship between antioxidant activity and TPC is frequently reported [11,27,29,35,52,53,67,82,83]. Moreover, individual phenolics also had higher levels overall in spring and summer. Phenolic compounds are powerful antioxidants that play a fundamental role against oxidative stress, not only physiologically in plants but also pharmacologically in terms of human health. They can protect cellular components against oxidative deterioration, limiting the onset of oxidative stress-associated diseases [84,85].

**Figure 7:**
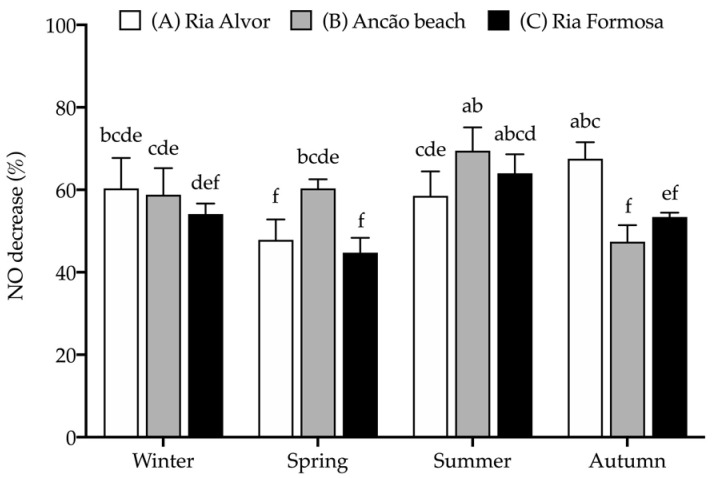
Seasonal and spatial anti-inflammatory activity (% NO decrease) of *C. edulis* (Hottentot-fig) extracts (100 μg/mL). L-Name (200 μg/mL) was used as a positive control (NO decrease: 73.4 ± 4.3%^a^). Values represent the mean ± SD (*n* = 6). Different letters represent significant differences, including the positive control (*p* < 0.05).

The extracts were further assessed for their anti-inflammatory activity by determining their capacity to decrease nitric oxide (NO) production when stimulating RAW 264.7 macrophages to produce NO (with bacterial lipopolysaccharide, LPS) in a chronic inflammatory state. The effect of seasonal and spatial variations on the anti-inflammatory activity of Hottentot-fig extracts is represented in Figure 7.

Hottentot-fig extracts (tested at 100 μg/mL) were able to reduce NO production and, although no clear tendency can be depicted, a higher percentage of NO decrease was registered in summer in site B (69.5%) and in autumn in site A (67.5%), with similar activity to that of the positive control, L-Name (73.4%, tested at 200 μg/mL). Additionally, all extracts were able to reduce NO production by more than 50%, except the extracts of samples from sites A and C in spring and site B in autumn (44.7–47.9% NO decrease). The anti-inflammatory activity of Hottentot-fig had previously been reported by Garcia-Oliveira et al. [14] in a hydro-ethanolic extract that inhibited NO by 50% at 237.9 ± 5.8 μg/mL (EC_50_ value), which can be considered less active than has presently been demonstrated. Of the phenolic compounds identified in Hottentot-fig extracts (Table 4), all seven of them have recognized anti-inflammatory activity, as described in the literature [65,66,70,74,77,79,80], which potentially explains the results that were obtained. Of these, chlorogenic acid (5-caffeoylquinic acid) was also identified by Garcia-Oliveira et al. [14] in their Hottentot-fig samples. Nevertheless, as previously stated, not all compounds in the extracts were identified; therefore, other molecules in the samples could also have contributed to the anti-inflammatory effects that were observed.

**Figure 8:**
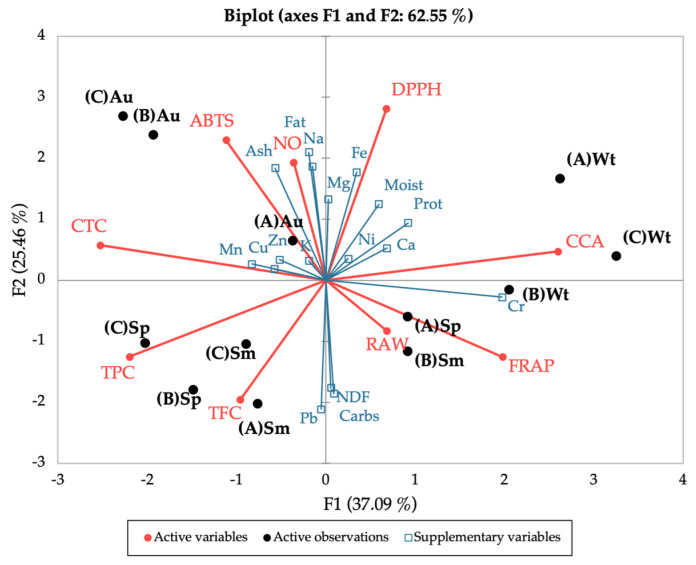
Principal Component Analysis of the seasonal and spatial bioactivities (DPPH, ABTS, NO, FRAP, CCA, RAW) and polyphenolic contents (TPC, TFC, CTC) of *C. edulis* (Hottentot-fig) extracts, coupled with the nutritional profile (proximate composition and mineral content) as supplementary variables. (A) Ria Alvor, (B) Ancão beach, (C) Ria Formosa; Wt: winter, Sp: spring, Sm: summer, Au: autumn.

Lastly, the seasonal and spatial variation of the bioactivities and polyphenolic contents, together with the nutritional profile, were correlated using Principal Component Analysis (PCA) (Figure 8), to uncover a pattern for the observed results. The PCA discriminated between seasons, placing spring and summer in the lower quadrants and autumn and winter in the upper quadrants (except site B in winter); this distribution in the vertical F2 axis accounts for 25.5% of the variation. Autumn and winter were also clearly separated, the first to the left and the latter to the right of the PCA, while spring and summer were evenly distributed; this horizontal distribution (F1) accounts for 37% of the variation. Overall, this placing of seasons by the PCA confirms the closer link between spring and summer, and autumn and winter, as portrayed in the nutritional, chemical, and bioactivity profiles. Of note, spring and summer seem to have a greater influence from the polyphenolic groups (TPC and TFC) and autumn (site A) from most minerals.

## 3. Materials and Methods

### 3.1. Chemicals

All chemicals were of analytical grade. The DPPH, ABTS, and NO radicals, the positive controls, BHA (butylated hydroxyanisole), ascorbic acid, and EDTA (ethylenediaminetetraacetic acid), the standards gallic acid, quercetin, and catechin, the commercial standards for the HPLC-DAD analysis (caffeic, chlorogenic, coumaric, cinnamic, 3,4-dihydroxybenzoic, ferulic, gallic, gentisic, 3-hydroxybenzoic, 4-hydroxybenzoic, salicylic, syringic, and vanillic acids, along with catechin hydrate, epicatechin, flavone, 4-hydroxybenzaldehyde, luteolin-7-*O*-glucoside, naringenin-7-glucoside, and rutin), and the chemicals aluminum chloride, copper sulfate, DMACA (4-dimethylaminocinnamaldehyde), ferrozine, Folin–Ciocalteu (F–C) reagent, L-Name (Nω-nitro-L-arginine methyl ester hydrochloride), LPS (lipopolysaccharides) from *Escherichia coli* O111:B4, MTT (3-(4,5-dimethylthiazol-2-yl)-2,5-diphenyltetrazolium bromide), phosphoric acid, pyrocatechol violet, sodium nitrite, sulphanilamide, and NED (N-(1-naphthyl) ethylenediamine dihydrochloride) were purchased from Merck Life Sciences (Sintra, Portugal). Dimethyl sulfoxide (DMSO), acetone, methanol, and chloroform were provided by Enzymatic (Santo Antão do Tojal, Portugal). Additional reagents/solvents were obtained from VWR International (Carnaxide, Portugal).

### 3.2. Sample Collection, Processing, and Extraction

Hottentot-fig’s aerial parts (leaves, shoots, and flowers when present) (voucher code XBH26, XtremeBio laboratory herbarium, Faro, Portugal) were harvested throughout the year (2020; meteorological values are given in Table 2) in winter (January), spring (May), summer (July), and autumn (November), in 3 locations in southern Portugal: (A) the Ria de Alvor lagoon near Portimão (37°07′34.6″ N 8°35′54.6″ W), (B) the Ancão beach near Loulé (37°01′54.0″ N 8°02′12.1″ W), and (C) the Ria Formosa lagoon near Faro (37°01′02.0″ N 7°59′39.3″ W), as represented in Figure 2. Collected samples were placed in a ventilated oven at 40 °C until completely dry, ground into powder, and stored at −20 ode XBH26, XtremeBio laboratory herbarium, Faro, PC until further processing and/or analysis.

Samples of dried biomass were extracted with aqueous acetone (80%; 1:40, *w*/*v*) for 24 h under stirring, at room temperature, filtered (filter paper Whatman grade 4), and concentrated at 40 °C under reduced pressure in a rotary evaporator (R-210, Buchi Labortechnik AG, Flawil, Switzerland). The dried extracts were redissolved at 25 mg/mL in DMSO and stored at −20 °C until needed.

### 3.3. Nutritional Profiling

#### 3.3.1. Proximate Composition

Freshly collected biomass was dried in a ventilated oven (105 °C, 16 h) to determine moisture content. Previously dried samples were analyzed for contents: of ash, by incineration (600 °C, 2 h) in a muffle furnace [86]; of crude protein, by assessing the total nitrogen (CHN Elemental Analyzer Vario EL III) and following the macro-Kjeldahl method (N × 6.25) [87]; of total lipids (crude fat), using a modified Bligh and Dyer method [88]; of total carbohydrates, calculated by difference. The results are conveyed as g/100 g DW. Neutral detergent fiber (NDF) content was assessed following the directive of the International Organization for Standardization (ISO), used to analyze animal feedstuffs (ISO 16472:2006), and the results are expressed as g/100 g DW.

#### 3.3.2. Mineral Composition

Dried biomass was also assessed for mineral content: samples were digested with a Microwave Digestion System (Discover SP-D 80, CEM Corp., Matthews, NC, USA) with 67% nitric acid (4 min ramp temperature to 200 °C, holding for 3 min), diluted with ultra-pure water (1:10), and analyzed with a Microwave Plasma-Atomic Emission Spectrometer (MP- AES; Agilent 4200 MP-AES, Agilent, Victoria, Australia), as described by Pereira et al. [89]. The results are conveyed as g/100 g DW for macro and as mg/100 g DW for trace elements.

### 3.4. Chemical Profiling

#### 3.4.1. Contents of Total Phenolics (TPC), Flavonoids, and Condensed Tannins (CTC)

The total phenolic (TPC), flavonoid (TFC), and condensed tannin (CTC) contents of the extracts were assessed via colorimetric assays, as fully described by Oliveira et al. [27]. The Folin–Ciocalteu reagent was used for TPC determination, measuring the absorbance at 725 nm (Biochrom EZ Read 400, Santa Clara, CA, USA), and using gallic acid as the standard for the calibration curve (1 μg/mL–1 mg/mL, *R*^2^ = 0.999). The aluminum chloride (AlCl_3_) assay was used in TFC evaluation, measuring the absorbance at 415 nm, and using quercetin as standard (1 μg/mL–1 mg/mL, *R*^2^ = 0.999). The DMACA method was used in CTC assessment, measuring absorbance at 640 nm, and using catechin as standard (1 μg/mL–1 mg/mL, *R*^2^ = 0.998). The results are expressed as mg of standard equivalents (GAE, QE, and CE, correspondingly) per g of extract DW.

#### 3.4.2. Phenolic Profile via High-Performance Liquid Chromatography-Diode Array Detection (HPLC-DAD)

Extracts at 10 mg/mL (90% ultrapure water + 10% methanol) were analyzed by High-Performance Liquid Chromatography-Diode Array Detection (HPLC-DAD; Agilent 1100 Series LC system, Germany), constituted by a vacuum degasser (G1322A), quaternary pump (G1311A), autosampler (G1313A), thermostatted column compartment (G1316A), and diode array detector (G1315B). Data acquisition and instrumental control were performed with LC3D ChemStation software (version Rev.A.10.02[1757], Agilent Technologies). The analyses were performed with a Mediterranea Sea 18 column of 15 × 0.21 cm^2^, with a 5 μm particle size (Teknokroma, Spain). The mobile phase comprised a mixture of methanol (solvent A) and 2.5% acetic acid aqueous solution (solvent B), with the following gradient: 0–5 min: 10% A, 5–10 min: 10–30% A, 10–40 min: 30–90% A, 40–45 min: 90% A, 45–55 min: 90–10% A, and 55–60 min: 10% A. The flow used was 0.35 mL/min and the injection volume was 20 μL, with a draw speed of 200 μL/min. The detector was set at 210, 280, 320 (used for quantification), and 350 nm. For identification purposes, the retention parameters of each assay were compared with the standard controls and the peak purity with the UV-visible spectral reference data. The different compound levels were extrapolated from the calibration standard curves. Commercial standards of caffeic, chlorogenic, coumaric, cinnamic, 3,4-dihydroxybenzoic, ferulic, gallic, gentisic, 3-hydroxybenzoic, 4-hydroxybenzoic, salicylic, syringic, and vanillic acids, and catechin hydrate, epicatechin, flavone, 4-hydroxybenzaldehyde, luteolin-7-*O*-glucoside, naringenin-7-glucoside, and rutin were prepared in methanol (1 mg/L) and diluted with ultrapure water to the chosen concentrations.

### 3.5. Bioactivity Profiling

Extracts were tested in serial dilution concentrations (from 10–0.01 mg/mL) to allow calculation of the half-maximal effective concentrations (EC_50_ values). Absorbances were measured using a microplate reader (Biochrom EZ Read 400, Santa Clara, CA, USA). Results were calculated as an activity percentage (%) in relation to a negative control (the solvent in redissolved samples), except for the ferric-reducing antioxidant power (FRAP) assay that was in relation to the positive control, and were expressed as EC_50_ values (mg/mL) whenever possible.

#### 3.5.1. In Vitro Antioxidant Activity

The extracts’ antioxidant properties were tested by radical-based methods, namely, scavenging activity toward the radicals DPPH• (1,1-diphenyl-2picrylhydrazyl), ABTS•+ (2,2′-azino-bis(3-ethylbenzothiazoline-6-sulfonic acid)), and NO (nitric oxide), and by metal-related methods, specifically, their ferric-reducing antioxidant power (FRAP) and their ability to chelate copper (CCA) and iron (ICA). BHA was used as a positive control for the DPPH•, ABTS•+, and FRAP assays, ascorbic acid was used for the NO method, and EDTA for the CCA and ICA methods. The assays have been described thoroughly by Oliveira et al. [27].

#### 3.5.2. In Vitro Anti-Inflammatory Activity

The extracts’ anti-inflammatory properties were tested using lipopolysaccharide (LPS) to stimulate the RAW 264.7 macrophages to produce nitric oxide (NO), as described by Rodrigues et al. [90]. The murine leukemic macrophage cell line (RAW 264.7) was kindly provided by the Mountain Research Center (CIMO, Bragança Polytechnic Institute, Portugal). Cells were cultured in RPMI medium, supplemented with 10% heat-inactivated fetal bovine serum, 1% L-glutamine (2 mM), and 1% penicillin (50 U/mL)/streptomycin (50 μg/mL), and were kept at 37 °C in a 5% CO_2_ humidified atmosphere. First, cells seeded at 10 × 10^3^ cells/well were incubated for 24 h with extracts at 100 μg/mL (diluted in culture medium) to evaluate cellular viability using the MTT assay. Non-treated cells in a culture medium containing DMSO (0.4%, in the same proportions as in the extracts) were used as a negative control. Once established that extracts at 100 μg/mL allowed more than 80% of cell viability (non-cytotoxic), they were incubated for 24 h with cells seeded at 2.5 × 10^5^ cells/well (in a serum- and phenol-free medium) and LPS (25 μg/mL). L-Name was used as a positive control. The Griess method was followed to determine the NO production, using sodium nitrite as a standard in the calibration curve. Non-treated, LPS-stimulated cells, in a culture medium containing DMSO (0.4%), were used as the negative control.

### 3.6. Statistical Analysis

All experiments were performed in triplicate. The results are expressed as mean ± standard deviation (SD). GraphPad Prism 8.4.3 for Mac (GraphPad Software, San Diego, CA, USA) was used to curve fit data and obtain EC_50_ values. Statistical differences (*p* < 0.05), analyzed using the XLSTAT trial version for Mac (Addinsoft 2023, New York, NY, USA), were assessed with a one-way analysis of variance (ANOVA), followed by the post hoc Tukey multiple comparison test. If data parametricity did not prevail, the Kruskal–Wallis and Dunn post hoc tests were used. Agglomerative Hierarchical Clustering (AHC) and Principal Component Analysis (PCA) were also assessed using XLSTAT.

## 4. Conclusions

This work represents the first attempt to study the seasonal and spatial variations of the nutritional, chemical, and bioactivity profiles of Hottentot-fig, in a comparative analysis of three different locations throughout four seasons. The Hottentot-fig’s aerial parts showed an adequate nutritional profile, akin to those of regularly consumed vegetables and edible halophytes, representing a good supply of carbohydrates, protein, and minerals, with a tendency for higher concentrations of the most relevant minerals and protein in autumn and winter, and in plants from sites A and B. Its extracts also showed that this species can be a rich source of phenolic compounds, particularly luteolin-7-*O*-glucoside, salicylic and coumaric acids, with antioxidant and anti-inflammatory properties, with emphasis on spring and summer as the optimal seasons for collection, although without there being any significant discrimination of locations. Optimized exploration of the discarded plant material may depend on the intended purposes of Hottentot-fig’s use, as the results point to a more balanced nutritional profile in autumn and winter while in spring and summer, the chemical and bioactivity profiles gain more relevance. Additionally, spatial variability should be considered, given its observed impact on nutrient contents. Overall, the usually wasted material of this invasive plant should be appraised as a proper nutritional source with potential biotechnological applications in the food and nutraceutical industries, namely, as an ingredient for value-added, preservative, and/or functional food products.

## Figures and Tables

**Table 1:** Seasonal and spatial nutritional profile of *C. edulis* (Hottentot-fig) aerial parts: proximate composition (g/100 g biomass DW (dry weight)) and mineral content (mg/100 g DW).

Location	(A): Ria de Alvor Lagoon					(B): Ancão Beach					(C): Ria Formosa Lagoon			
Season	Winter	Spring	Summer	Autumn		Winter	Spring	Summer	Autumn		Winter	Spring	Summer	Autumn
Proximate composition (g/100 g DW)														
Moisture	93.3 ± 0.4 ^ab^	90.4 ± 0.9 ^bc^	89.2 ± 0.6 ^c^	94.3 ± 0.5 ^a^		88.5 ± 1.2 ^cd^	87.9 ± 2.4 ^cd^	85.2 ± 0.2 ^d^	88.9 ± 0.4 ^c^		89.4 ± 0.8 ^c^	89.2 ± 1.9 ^c^	87.5 ± 1.0 ^cd^	88.5 ± 0.0 ^cd^
Ash	22.7 ± 0.6 ^bc^	21.6 ± 0.6 ^bc^	23.9 ± 0.2 ^b^	27.5 ± 0.2 ^a^		23.7 ± 0.3 ^b^	18.6 ± 0.7 ^d^	19.0 ± 0.8 ^d^	23.0 ± 0.1 ^bc^		21.8 ± 0.4 ^bc^	23.3 ± 0.2 ^b^	20.7 ± 0.2 ^cd^	27.0 ± 2.5 ^a^
Crude protein	6.2 ± 0.5 ^a^	3.8 ± 0.3 ^c^	3.2 ± 0.3 ^cd^	5.0 ± 0.5 ^b^		2.4 ± 0.4 ^defg^	1.8 ± 0.2 ^efg^	1.8 ± 0.2 ^fg^	1.6 ± 0.2 ^g^		2.6 ± 0.5 ^defg^	2.6 ± 0.4 ^def^	2.8 ± 0.3 ^de^	3.2 ± 0.2 ^cd^
Crude fat	1.0 ± 0.0 ^cd^	1.2 ± 0.0 ^abcd^	0.8 ± 0.1 ^fg^	1.3 ± 0.0 ^ab^		1.1 ± 0.1 ^bcd^	1.0 ± 0.1 ^def^	1.0 ± 0.0 ^cde^	1.3 ± 0.1 ^a^		0.8 ± 0.1 ^efg^	0.8 ± 0.1 ^g^	0.6 ± 0.1 ^g^	1.2 ± 0.1 ^abc^
Carbohydrates	70.0 ± 0.4	73.4 ± 0.4	72.0 ± 0.2	66.2 ± 0.3		72.8 ± 0.3	78.5 ± 0.4	78.2 ± 0.5	74.1 ± 0.2		74.8 ± 0.4	73.3 ± 0.2	75.9 ± 0.2	68.6 ± 1.5
Fiber, NDF	22.3	24.6	26.0	20.3		17.8	19.2	24.9	17.1		18.9	22.1	23.1	20.2
Minerals—Macro elements (g/100 g DW)														
Ca	3.7 ± 0.0 ^bc^	3.5 ± 0.0 ^cd^	2.7 ± 0.0 ^fgh^	2.9 ± 0.0 ^f^		4.3 ± 0.1 ^a^	3.4 ± 0.1 ^d^	3.3 ± 0.1 ^de^	3.7 ± 0.0 ^b^		2.6 ± 0.0 ^gh^	3.1 ± 0.1 ^e^	2.5 ± 0.0 ^h^	2.8 ± 0.1 ^fg^
K	2.0 ± 0.0 ^d^	1.7 ± 0.1 ^e^	1.5 ± 0.0 ^f^	1.6 ± 0.0 ^ef^		0.5 ± 0.0 ^g^	0.6 ± 0.0 ^g^	0.6 ± 0.0 ^g^	0.3 ± 0.0 ^h^		2.6 ± 0.1 ^c^	3.0 ± 0.1 ^a^	2.9 ± 0.0 ^ab^	2.8 ± 0.0 ^b^
Mg	0.8 ± 0.0 ^d^	0.7 ± 0.0 ^ef^	0.8 ± 0.0 ^d^	0.8 ± 0.0 ^d^		0.6 ± 0.0 ^g^	0.5 ± 0.0 ^h^	0.7 ± 0.0 ^fg^	0.8 ± 0.0 ^de^		1.1 ± 0.0 ^a^	0.9 ± 0.0 ^c^	0.9 ± 0.0 ^c^	1.0 ± 0.0 ^b^
Na	3.3 ± 0.1 ^d^	2.5 ± 0.0 ^f^	3.9 ± 0.1 ^c^	4.8 ± 0.1 ^a^		3.8 ± 0.2 ^c^	2.3 ± 0.0 ^f^	2.4 ± 0.1 ^f^	4.2 ± 0.1 ^b^		2.9 ± 0.1 ^e^	2.3 ± 0.1 ^f^	2.3 ± 0.0 ^f^	3.8 ± 0.0 ^c^
Trace elements (mg/100 g DW)														
Fe	5.0 ± 0.1 ^a^	2.5 ± 0.0 ^f^	2.3 ± 0.0 ^f^	3.3 ± 0.1 ^de^		4.8 ± 0.4 ^ab^	3.5 ± 0.0 ^cde^	4.1 ± 0.6 ^bc^	5.3 ± 0.3 ^a^		3.0 ± 0.2 ^ef^	3.0 ± 0.0 ^ef^	3.9 ± 0.0 ^cd^	3.6 ± 0.1 ^cde^
Mn	2.9 ± 0.1 ^g^	2.3 ± 0.1 ^g^	2.8 ± 0.0 ^g^	2.5 ± 0.0 ^g^		10.6 ± 0.2 ^d^	7.2 ± 0.2 ^f^	8.4 ± 0.2 ^e^	12.1 ± 0.1 ^c^		16.3 ± 1.1 ^b^	24.8 ± 0.2 ^a^	16.2 ± 0.3 ^b^	15.6 ± 0.1 ^b^
Zn	2.0 ± 0.1 ^ef^	1.5 ± 0.0 ^g^	1.1 ± 0.0 ^h^	1.3 ± 0.0 ^gh^		2.3 ± 0.1 ^e^	1.9 ± 0.1 ^f^	2.1 ± 0.1 ^ef^	3.1 ± 0.0 ^d^		7.4 ± 0.2 ^b^	8.7 ± 0.1 ^a^	7.2 ± 0.1 ^b^	6.1 ± 0.1 ^c^
Cu	0.3 ± 0.0 ^c^	0.3 ± 0.0 ^c^	0.2 ± 0.0 ^d^	0.4 ± 0.0 ^c^		0.3 ± 0.0 ^c^	0.3 ± 0.0 ^c^	0.4 ± 0.0 ^c^	0.3 ± 0.0 ^c^		0.6 ± 0.0 ^b^	0.6 ± 0.0 ^b^	0.9 ± 0.0 ^a^	0.6 ± 0.0 ^b^
Cr	0.1 ± 0.0 ^b^	0.0 ± 0.0 ^de^	0.0 ± 0.0 ^de^	0.1 ± 0.0 ^c^		0.1 ± 0.0 ^b^	0.0 ± 0.0 ^cd^	0.1 ± 0.0 ^a^	0.0 ± 0.0 ^cd^		0.1 ± 0.0 ^b^	0.0 ± 0.0 ^de^	0.0 ± 0.0 ^cde^	0.0 ± 0.0 ^e^
Ni	0.2 ± 0.0 ^ab^	0.1 ± 0.0 ^efg^	0.1 ± 0.0 ^ef^	0.2 ± 0.0 ^de^		0.1 ± 0.0 ^gh^	0.1 ± 0.0 ^h^	0.1 ± 0.0 ^fg^	0.1 ± 0.0 ^h^		0.2 ± 0.0 ^cd^	0.2 ± 0.0 ^ab^	0.2 ± 0.0 ^a^	0.2 ± 0.0 ^bc^
Cd	<LOQ ^1^	<LOQ ^1^	<LOQ ^1^	<LOQ ^1^		<LOQ ^1^	<LOQ ^1^	<LOQ ^1^	<LOQ ^1^		<LOQ ^1^	<LOQ ^1^	<LOQ ^1^	<LOQ ^1^
Pb	0.1 ± 0.0 ^d^	0.2 ± 0.0 ^abcd^	0.4 ± 0.1 ^a^	0.4 ± 0.1 ^a^		0.2 ± 0.0 ^bcd^	0.2 ± 0.0 ^cd^	0.3 ± 0.0 ^abc^	0.1 ± 0.0 ^cd^		0.3 ± 0.0 ^abc^	0.3 ± 0.0 ^ab^	0.3 ± 0.0 ^abc^	0.1 ± 0.0 ^d^

Values represent mean ± standard deviation (SD) (*n* = 3). NDF: neutral detergent fiber; DW: dry weight; LOQ: limit of quantification. LOQs: ^1^ Cd = 0.02 mg/100 g DW. For each line, different letters represent significant differences (*p* < 0.05).

**Table 2:** Meteorological values of the mean temperatures (minimum and maximum) and total precipitation registered in the collection months (source: IPMA 2020) [28].

Season	Month	$\bar{X}$ min. temp. *	$\bar{X}$ max. temp. *	Total Precipitation
Winter	January	8.9 °C	16.6 °C	29.6 mm
Spring	May	17.0 °C	24.7 °C	37.5 mm
Summer	July	21.6 °C	30.3 °C	0.0 mm
Autumn	November	13.7 °C	20.0 °C	155.8 mm

* $\bar{X}$ min. temp.: mean of the minimum temperature; $\bar{X}$ max. temp.: mean of the maximum temperature.

**Table 3:** Seasonal and spatial phenolic profile (mg/g extract DW) of *C. edulis* (Hottentot-fig) extracts by HPLC-DAD.

Peak	RT	Compound	(A): Ria de Alvor Lagoon				(B): Ancão Beach				(C): Ria Formosa Lagoon			
n	(min)		Winter	Spring	Summer	Autumn	Winter	Spring	Summer	Autumn	Winter	Spring	Summer	Autumn
*Phenolic acids*														
Hydroxybenzoic acids														
1	5.9	Gentisic acid	0.03	0.07	0.09	0.03	0.04	0.07	0.01	0.03	0.07	0.09	0.05	0.00
6	14.6	Salicylic acid	1.68	1.56	2.14	1.35	1.31	1.44	2.34	1.32	1.05	1.78	0.88	1.05
Hydroxycinnamic acids														
2	9.6	Chlorogenic acid	0.24	0.33	0.30	0.24	0.23	0.25	0.11	0.04	0.17	0.25	0.12	0.13
3	10.2	Caffeic acid	0.51	0.73	0.48	0.40	0.30	0.35	0.29	0.28	0.21	0.31	0.23	–
4	13.4	Coumaric acid	0.35	0.34	0.49	–	0.30	0.38	0.31	0.30	0.34	0.38	0.38	0.45
5	14.3	Ferulic acid	0.10	0.13	0.12	0.14	0.13	0.17	–	0.24	0.09	0.24	0.22	0.12
*Flavonoids*														
Flavones														
7	17.9	Luteolin-7-*O*-glucoside	0.43	0.55	0.42	0.43	1.56	0.76	1.37	2.68	2.10	2.36	1.26	2.44
		Σ phenolics	3.34	3.71	4.04	2.59	3.87	3.42	4.43	4.89	4.03	5.41	3.14	4.19

RT: retention time; –: not detected. Peaks are numbered according to retention time.

**Table 4:** Seasonal and spatial antioxidant activity (EC_50_ values, mg/mL) of *C. edulis* (Hottentot-fig) extracts: radical scavenging on DPPH•, ABTS•+, and NO radicals, ferric = reducing antioxidant power (FRAP), and metal-chelating activities on copper (CCA) and iron (ICA).

Location	Season	DPPH•	ABTS•+	NO	FRAP	CCA	ICA
(A): Ria de Alvor	Winter	0.22 ± 0.00 ^d^	0.08 ± 0.01 ^abc^	2.17 ± 0.02 ^f^	0.50 ± 0.03 ^f^	4.23 ± 0.18 ^f^	>10
	Spring	0.07 ± 0.01 ^ab^	0.11 ± 0.01 ^bc^	0.26 ± 0.02 ^ab^	0.29 ± 0.03 ^d^	3.50 ± 0.20 ^e^	>10
	Summer	0.08 ± 0.01 ^ab^	0.09 ± 0.01 ^abc^	1.24 ± 0.01 ^d^	0.56 ± 0.02 ^g^	2.68 ± 0.15 ^d^	>10
	Autumn	0.18 ± 0.02 ^c^	0.19 ± 0.02 ^d^	0.85 ± 0.07 ^c^	0.12 ± 0.00 ^ab^	2.57 ± 0.21 ^d^	>10
(B): Ancão beach	Winter	0.17 ± 0.00 ^c^	0.08 ± 0.02 ^abc^	0.41 ± 0.03 ^b^	0.41 ± 0.02 ^e^	4.88 ± 0.31 ^g^	>10
	Spring	0.06 ± 0.01 ^ab^	0.06 ± 0.00 ^a^	0.21 ± 0.01 ^a^	0.18 ± 0.00 ^bc^	1.99 ± 0.02 ^bc^	>10
	Summer	0.10 ± 0.00 ^b^	0.11 ± 0.01 ^bc^	0.98 ± 0.03 ^c^	0.56 ± 0.01 ^g^	3.38 ± 0.04 ^e^	>10
	Autumn	0.29 ± 0.02 ^e^	0.25 ± 0.03 ^e^	1.67 ± 0.13 ^e^	0.12 ± 0.01 ^ab^	2.46 ± 0.20 ^d^	>10
(C): Ria Formosa	Winter	0.22 ± 0.01 ^d^	0.12 ± 0.01 ^c^	0.16 ± 0.01 ^a^	0.64 ± 0.05 ^h^	4.73 ± 0.09 ^g^	>10
	Spring	0.05 ± 0.00 ^a^	0.07 ± 0.00 ^ab^	0.18 ± 0.01 ^a^	0.21 ± 0.02 ^c^	1.84 ± 0.08 ^b^	>10
	Summer	0.09 ± 0.00 ^ab^	0.05 ± 0.00 ^a^	1.25 ± 0.02 ^d^	0.44 ± 0.01 ^ef^	2.51 ± 0.04 ^d^	>10
	Autumn	0.18 ± 0.01 ^c^	0.68 ± 0.02 ^g^	1.66 ± 0.09 ^e^	0.10 ± 0.00 ^a^	2.37 ± 0.14 ^cd^	>10
Positive controls	BHA	0.60 ± 0.03 ^f^	0.33 ± 0.02 ^f^		0.16 ± 0.01 ^abc^		
	EDTA					0.16 ± 0.00 ^a^	0.03 ± 0.00 ^a^
	Asc. acid			1.71 ± 0.02 ^e^			

Values represent the mean ± SD of at least three experiments performed in triplicate (*n* = 9). For each assay (column), different letters represent significant differences (*p* < 0.05).

## Data Availability

The dataset is available upon request from the corresponding author.
